# Cost-effectiveness of using a motion-sensor biofeedback treatment approach for the management of sub-acute or chronic low back pain: economic evaluation alongside a randomised trial

**DOI:** 10.1186/s12891-016-1371-6

**Published:** 2017-01-17

**Authors:** Terry Haines, Kelly-Ann Bowles

**Affiliations:** Allied Health Research Unit & Physiotherapy Department, School of Primary Health Care, Monash University and Monash Health, Kingston Rd, Cheltenham, 3192 Australia

**Keywords:** Low back pain, Economic evaluation, Cost-effectiveness, Randomized trial

## Abstract

**Background:**

Low back pain is a common and costly condition internationally. There is high need to identify effective and economically efficient means for managing this problem. This study aimed to explore the cost-effectiveness of a novel motion-sensor biofeedback treatment approach in addition to guidelines-based care compared to guidelines-based care alone, from a societal perspective over a 12 month time horizon.

**Method:**

This was an incremental cost-effectiveness analysis conducted concurrently with a pilot, cluster randomized controlled trial. Health care resource use was collected using daily diaries and patient-self report at 3, 6 and 12 month follow-up assessments. Productivity was measured using industry classifications and participant self-reporting of ability to do their normal work with their present pain. Clinical effect was measured using the Patient Global Impression of Change measured at the 12 month follow-up assessment. Data were compared between groups using linear regression clustered by recruitment site. Bootstrap resampling was used to generate a visual representation of the 95% confidence interval for the incremental cost-effectiveness estimate. Two, one-way sensitivity analyses were undertaken to examine the robustness of findings to key assumptions.

**Result:**

There were *n* = 38 participants in the intervention group who completed the 12 month assessment and *n* = 45 in the control. The intervention group had greater use of trial-related medical and therapy resources [$477 per participant (95% CI: $447, $508)], but lower use of non-trial medical and therapy resources [$-53 per participant (95% CI: $-105, $-0)], and a greater improvement in productivity [$-5123 per participant (95% CI: $-10,174, $-72)]. Overall, the intervention dominated with a saving of $478,100 and an additional 41 participants self-rating as being very or much improved compared to the control. There was >99% confidence in this finding of dominance in both the primary and sensitivity analyses.

**Conclusions:**

The motion-sensor biofeedback treatment approach in addition to guidelines- based care appears to be both more clinically effective and economically efficient than guidelines- based care alone. This approach appears to be a viable means to manage low back pain and further research in this area should be a priority.

**Trial registration:**

The randomised trial this research was based upon was prospectively registered on March 25th 2009 with the Australian New Zealand Clinical Trials Registry: ACTRN12609000157279.

## Background

Low back pain presents a tremendous cost to developed nations internationally [1]. These costs can be incurred by patients directly, and by society as a whole through increased use of publicly subsidized health services and reduced paid occupational activity [2]. Loss of productivity (in both paid and unpaid occupations) has been identified as a key driver of these costs [3]. There is need to understand how this burden of disease can be minimised.

There are many interventions that have been argued as being beneficial for management of low back pain. Some approaches, such as spinal surgery, come at substantial cost to patients and/or health care providers. Other options, such as physical therapy, are less costly to deliver but the comparative cost-effectiveness of these approaches remains relatively unknown [4]. In the absence of robust evidence demonstrating the relative cost effectiveness of different treatment options, clinicians are left only with evidence examining the effectiveness of different treatments to guide their selection. There are conflicting results present in this literature and best practice guidelines now make somewhat generic recommendations regarding evaluation and management approaches for clinicians treating low back pain patients [5–7]. A review of the cost-effectiveness of these approaches has identified that interdisciplinary rehabilitation, exercise, acupuncture, spinal manipulation and cognitive-behavioural therapy may all be cost-effective for management of sub-acute or chronic low back pain [8].

Recent advances in technology have permitted development of new approaches to manage low back pain. One such approach has been the use of a motion-sensor biofeedback systems. Biofeedback has been used for the management of low back pain as far back as the 1980’s with some promising short-term results [9]. These interventions however were largely restricted to laboratory settings as the equipment was not readily portable. Recent advances in the portability of this technology now allow patients to understand how their low back moves, and have their posture monitored while performing everyday activities so that clinicians can receive a detailed log of how the patient moved during the day and/or night. Using the data from the ambulatory monitoring session, the device can be personalised to notify or remind patients of optimal movements and postures based on their own condition. A recent multicentre, cluster-randomised, placebo-controlled, pilot clinical trial reported a significant and sustained improvement in pain and activity limitation that persisted several months after the initial biofeedback treatment sessions were completed using this technology [10].

Adoption of new technologies in clinical practice should be driven by evidence of both efficacy and of economic efficiency [11]. There has been much recent comment on the spiralling costs of health care being driven, in part, by the increasing costs of service provision attributable to new technologies [12]. No economic evaluation of the use of modern motion-sensor biofeedback systems for the management of low back pain has previously been presented in the literature. However, the value of adding this treatment approach to conventional, guidelines based care needs to be established. This study aimed to explore the cost-effectiveness of a novel motion-sensor biofeedback treatment approach in addition to guidelines-based care compared to guidelines-based care alone, from a societal perspective over a 12 month time horizon.

## Methods

### Design

Incremental cost-effectiveness study conducted concurrently with a pilot, cluster randomised trial. The cluster randomised trial included eight recruiting sites randomised to either an intervention group consisting of motion-sensor biofeedback combined with “guidelines-based care” or a “guidelines-based care” only control group. Further details of this trial can be found in the trial report paper [10]. This economic evaluation took a societal perspective over a 12 month follow-up time horizon from the date of commencing participation in the trial.

### Participants and setting

This trial was conducted across eight sites (two hospitals, six outpatient primary care clinics) in Victoria, Australia. The participating clinicians were two physicians, four GPs and three physiotherapists, all with a special interest in musculoskeletal conditions. The medical practitioners had an average of 25.8 years post-graduate experience and the physiotherapists 19.0 years.

**Fig. 1:**
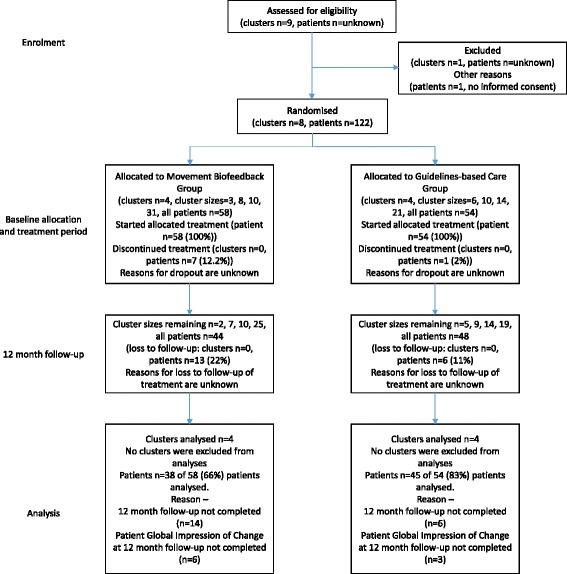
Participant flow through study

Patients approached for inclusion into this study needed to be aged between 18 and 65 years, have a primary complaint of low back pain or back-related leg pain, have an average pain intensity of three or more on a 0–10 scale, and an episode duration of at least 3 weeks. Patients were excluded from this study if they had surgery or another invasive procedure on their lumbar spine within the previous 12 months, if they were pregnant, had severe hearing impairment, had an implanted electrical medical device, had a known allergic skin reaction to tapes and plasters or any of a range of comorbid disorders including: neoplasm, infection, inflammatory or neurological disorder, fracture or other joint or medically-related disorders. The flow of participants is presented (Fig. 1).

### Randomisation

This was a cluster randomised trial where clinics were the unit of randomisation. Consequently, clinicians at each clinic delivered only one type of treatment. Patient recruitment occurred from each clinician’s usual patient flow and clinicians were not blind to treatment allocation. Randomisation was undertaken by allocating each of the three participating physiotherapy clinics to be randomly paired with one of the participating medical clinics, and the remaining two medical clinics formed a fourth and final pair. Each pair was arbitrarily given a number from 1 to 4, and each clinic given an arbitrary code (A or B) within each pair. These four numbered and paired codes, without clinic identification (blinded), were given to a researcher (TH) who generated a random number between 0.0 and 1.0 for Clinic A in each of the four pairs using Excel (Microsoft Corp, Redmond WA, USA). If the number was >0.5, Clinic A was assigned to be a Movement Biofeedback Group clinic and its paired Clinic B to be a Guidelines-based Care Group clinic. If the number was <0.5, the assignment direction was the reverse. This procedure resulted in one physician, one GP and two physiotherapists being randomised to the intervention (movement biofeedback) group and one physician, two GPs and one physiotherapist being randomised to the control (guidelines-based care only) group.

#### Funding

Funding for this study was equally provided by (i) a grant from the Department of Business and Innovation (Market Validation Program), Victorian Government, Australia, and (ii) dorsaVi P/L (the Australian company who manufactures the ViMove motion-sensor system used in this study). The Department of Business and Innovation helped in the governance of the trial. DorsaVi supplied the motion-sensor equipment and coordinated the trial, assisted by a contract research organisation (Kendle P/L, Oakleigh, Victoria, Australia). All data and trial-related documentation were independently audited by Paul L Clark and Associates (Beaumaris Victoria, Australia). The authors analysed the results and wrote this paper independently of both funders, and neither funder had any influence over how these data were presented and the conclusions reached.

### Description of treatments

#### All participants

All participants in this trial received advice on staying active and general self-management of back pain. This advice was based on the 2003 Australian National Health and Medical Research Council guidelines for the management of acute low back pain [13], and European guidelines for the management of chronic non-specific LBP [14] in the absence of similar Australian guidelines for chronic LBP. The participants could also have received whatever usual medical and physiotherapy care was deemed essential by their clinicians, and such guidelines-based [13, 14] co-interventions were noted. These treatments and advice were metered out over 6–8 sessions over a 10 week period at the commencement of the trial.

#### Intervention participants

The motion-sensor biofeedback system investigated in this research was the ViMove motion-sensor system (Version 5, dorsaVi.com). This system consists of: (i) two wireless motion-sensors that measure three-dimensional movement, movement velocity and acceleration, and orientation to gravity, (ii) two wireless surface electromyography (EMG) sensors that measure para-spinal muscle activation, (iii) a wireless recording device (approximately the size of a small mobile phone) that captures the sensor data, has a button that patients can push when an event occurs (such as an onset or increase in pain), an audio and vibration function that can be programmed to provide patient-specific biofeedback alerts, and (iv) a charging dock for these wireless devices. The system also has a comprehensive computer software application that clinicians use to observe movement characteristics in real-time, to download postural and movement data from the recording device captured during activities of daily living, to analyse these data with the use of graphics-rich reports, and to compare an individual’s movement pattern with their previous assessments or with reference values.

Participants in the intervention group had an individualised assessment including a physical examination and biomechanical movement analysis using the ViMove system to examine potential relationships between their movement or posture and their pain. The ViMove was worn both in the clinic and during the patient’s activities of daily living. The clinician then devised a patient-specific rehabilitation strategy designed to address any identified deficits in the patient’s pattern of lumbo-pelvic movement and/or posture. For example, output from the ViMove device could be used to differentiate pelvic tilt movement from lumbar spine flexion movement when a participant attempted to reach towards the ground. If movement limitation was identified and found to be largely attributable to limited lumbar spine flexion rather than pelvic tilt, then the lumbar spine flexion component could be targeted for treatment. Clinicians provided ‘live training’ in the clinic, where patients were instructed in how to alter their movement pattern(s) or posture using real-time on-screen biofeedback, while wearing the ViMove device. Clinicians could also program the ViMove to provide motion-sensor biofeedback alerts (audio ‘beeps’ and/or vibration of the wireless recording device) that would occur during 4- to 10- h periods during which participants wore the device at home. This biofeedback would prompt the patient when they ‘broke a rule’ that the clinician had programmed. The clinician could also prescribe specific exercises that supplemented the patient-specific movement biofeedback.

#### Control participants

Control participants received the care as described for “all participants” which included a combination of education and advice, exercises, imaging, manual therapy, medication and taping/bracing as previously described [10]. In addition, they wore the ViMove device 6 to 8 times over the 10-week treatment period to mitigate any potential placebo effects of wearing this equipment. Their clinicians were blocked via a software lock during the trial to any of the motion-sensor/EMG information collected. Patients in the control group were informed that the ViMove system was a measurement device.

### Measurements

A range of demographic data and patient outcome questionnaires, including the Fear Avoidance Beliefs Questionnaire, the Quadruple Visual Analogue Scale of pain intensity, the Roland-Morris Disability Questionnaire, the Patient Specific Functional Scale were collected at baseline and periodically during the trial. These measures have previously been reported on [10].

#### Measurement of costs

An estimate of productivity was developed using participant self-reported industry field (18 possible codes) based on gender-specific data provided by the Australian Bureau of Statistics [15]. The Australian Bureau of Statistics provides mean income (based on 2015 data) for these classifications. These industry estimates were pro-rated against the degree to which participants reported they could perform the duties of their occupation. An item from the Fear Avoidance Beliefs Questionnaire was used to gauge the degree to which back pain limited participation in their occupation [16]. This item was worded as “I cannot do my normal work with my present pain” and was scaled on a 0 to 6 scale with zero being “completely disagree”, three being “unsure” and six being “completely agree”.

Use of pain medications was estimated based on self-reported medication use captured at baseline and any change in medication use captured using the daily pain diary. The daily pain diary was scheduled for completion over the first 3 months of the trial. Medications to treat unrelated conditions (eg. heart disease) were not captured as these were assumed to have little variance over the follow-up period from baseline attributable to the management of back pain. Medication costs were valued using market prices based on the Australian Pharmaceutical Benefits Schedule and market rates for medications that are not subsidized under this scheme.

Use of imaging and general practitioner visits for back pain complaints were captured from participant self-report at the 3, 6, 9 and 12 month assessments. These costs were valued using market prices based on the Australian Medical Benefits Schedule (eg. item 63557 is $492.80 AUD for magnetic resonance image of lumbar spine). Use of medical or therapies (eg. physiotherapy, chiropractic) received for management of low back pain in addition to the intervention or control services provided were captured from participant self-report at the 3, 6, 9 and 12 month assessments. These services were valued using market prices based on locally advertised rates for private practitioner services (i.e. $77.95 AUD per visit). The use of medical services and therapies provided as a part of the intervention and control conditions were captured directly by project data collectors based on attendance at treatment sessions. These services were valued using the same local market prices ($77.95 AUD per visit). We added a cost per session for intervention group participants for having access to the ViMove system and the consumables required for its use. There is an annual fee of $5000 AUD per year for the software license and one set of equipment. In clinical practice, it may be feasible to use one set of equipment for up to 33 patients per year if applied in the same manner as in the randomised controlled trial (“in clinic” and “at home” monitoring with 6–8 sessions per patient). However, for this economic evaluation, we have conservatively estimated that one set of equipment could be used for ~12 patients in 1 year. This creates a “per-participant” cost of $416.67, which is approximately $60 per session if seven sessions are booked on average. DorsaVi specific adhesives were used to attach the motion analysis sensors and electromyography sensors in the trial. We have conservatively added a $20 per session cost to cover the price of adhesives and other consumables required to apply this equipment. This resulted in a total $80 cost per session per participant. However, electromyography sensors may not be required for all assessments subsequent to the initial assessment, which in real life would reduce the consumables cost to ~ $9 per session.

All costs were calculated in Australian dollars ($AUD) at 2015 values.

#### Measurement of outcome

The clinical outcome used for this economic evaluation was the Patient Global Impression of Change measured at the 12 month follow-up assessment [17]. This scale measures improvement relative to participant recall of their condition at baseline on a seven-point ordinal Likert scale (Very much improved, Much improved, Minimally improved, No change, Minimally worse, Much worse, Very much worse). The Patient Global Impression of Change has shown high levels of reliability and construct validity [18, 19].

### Procedure

Clinics and clinicians were recruited by staff administering the trial. Patients were then recruited by their treating clinicians. All participants provided written informed consent. Participants were provided with their respective treatments and data for the economic evaluation was collected via the baseline assessment, the daily pain diaries (first 3 months of trial) and assessments conducted with a research assistant blinded to group allocation at 3 months, 6 months and 1 year.

### Analyses

Measures of productivity and medication use for low back pain were calculated as change scores relative to baseline. These change scores were calculated over a 12 month follow-up time horizon using an area under the curve approach. Only participants who completed the final follow-up assessment (which included the only assessment of clinical outcome measure for this economic evaluation) were included in these analyses, making this a form of “complete case” analysis approach. If a participant missed a particular follow-up prior to the 12 month assessment, then the area under the curve approach was used to impute the missing data for change scores based on the adjacent assessment points. Measures of clinical visits and imaging for management of low back pain were calculated as absolute values consumed over the 12 month follow-up period. No participants reported use of hospitals or surgery for management of low back pain, thus these costs did not affect our estimate of direct health costs. A total cost figure was calculated for each participant, being the sum of productivity loss (or gain) over the 12 month follow-up period relative to baseline, the increase (or decrease) in medication use cost over the 12 month follow-up period relative to baseline, and the absolute cost of clinical visits outside of the trial therapy visits, imaging, and cost of clinical visits as a part of the trial protocol. The total cost figure was used to form the numerators in the incremental cost effectiveness ratio.

The dichotomous measure of effect used to form the denominator in the incremental cost effectiveness ratio was generated by merging participants who rated themselves as very much improved or much improved into one category, with all other responses being merged into the other category. The incremental cost effectiveness ratio was then able to be calculated as being:

1 $$ \mathrm{Incremental}\ \mathrm{cost}\ \mathrm{effectiveness}=\frac{\mathrm{Total}\;\mathrm{Cost}\;\left(\mathrm{Intervention}\;\mathrm{Group}\right)-\mathrm{Total}\;\mathrm{Cost}\left(\mathrm{Control}\;\mathrm{Group}\right)}{\%\mathrm{Very}\ \mathrm{or}\ \mathrm{Much}\ \mathrm{Improved}\left(\mathrm{Intervention}\ \mathrm{Group}\right)-\%\ \mathrm{Very}\ \mathrm{or}\ \mathrm{Much}\ \mathrm{Improved}\left(\mathrm{Control}\ \mathrm{Group}\right)} $$

The numerator of this ratio was calculated using linear regression analyses of the total cost variable so that the difference between groups in their geometric mean of the total cost variable could be calculated. We used Huber White “sandwich” variance estimators and clustered data based on the site from which the participant was recruited in keeping with best practice for analyses of cluster randomised trials (so that the units of analyses are equivalent to the units of randomisation) [20]. We used a similar approach to calculate the difference between groups in proportion of participants who were very or much improved according to their reported Patient Global Impression of Change. We used bootstrap resampling to calculate a 95% confidence ellipse to visually represent the uncertainty surrounding the incremental cost effectiveness estimate generated [21]. We conducted 2000 replications of the original sample, based on the original sample size that was collected within allocated groups. The analyses were repeated for each bootstrap replication, and the ensuing results plotted. Cost-effectiveness acceptability curve analyses were pursued to identify the probability that the intervention program was more effective and less costly from the societal perspective than the control program [22].

Two sensitivity analyses were pursued to examine the impact of key assumptions made for the primary analysis. First, we changed the threshold for dichotomization of our clinical outcome, so that we now combined those with a response of very much improved, much improved, or minimally improved into the one category. This is a justifiable sensitivity analysis to pursue as there are no universally accepted guidelines determining how much improvement in the Patient Global Impression of Change scale is necessary to be clinically significant. In the second sensitivity analysis, we excluded the cost of providing “trial” therapy to control group participants. This is a more controversial choice, though can be justified under the reasoning that those in the control group may not ordinarily have pursued the amount and duration of trial therapy sessions included in this trial in a real life context, yet still achieved the same clinical outcome. This sensitivity analysis intentionally has the effect of providing a more conservative estimate of the cost-effectiveness of the intervention.

## Results

There were *n* = 9 sites assessed for eligibility of which eight met study inclusion criteria and were recruited into the study. These sites recruited *n* = 112 participants of whom *n* = 38 (intervention) and *n* = 45 (control) completed the Patient Global Impression of Change at the final assessment. These numbers are lower than the total number who completed the final assessment in the pilot trial as the Patient Global Impression of Change was added to the study protocol after the first 11 patients were scheduled to have completed their 12 month follow-up. A description of participant demographics and their use of medication and productivity data at baseline is presented (Table 1). Other demographics of this sample have previously been reported [10]. The mean (sd) change in medication use and productivity costs, along with mean (sd) absolute costs for use of imaging, medical and therapy services (within the trial and external to the trial) over the follow-up period are presented (Table 2). Three participants in the intervention group had a total of four MRI scans of their lumbar spine compared to six participants in the control group each having one over the follow-up period. Thirteen participants in the intervention group had an additional 67 therapy or medical appointments for their back pain over the follow-up whereas twenty participants in the control group had an additional 88 therapy or medical appointments. We identified significant difference between groups in term of productivity changes (intervention group participants became more productive relative to baseline compared to control), in the resources consumed in trial based medical and therapy services (intervention group consumed more) and in resources consumed in non-trial based medical and therapy services (control group consumed more). Table 2 also presents the break-down of responses to the Patient Global Impression of Change scale across intervention and control groups where there were significant differences in favour of the intervention group.

**Table 1:** Participant demographics and baseline data from participants who completed the 12 month assessment

	Intervention	Control
*n*	38	45
Age	39 (14)	49 (12)
Gender	18 (47%) female	26 (58%) female
Pain intensity (QVAS) (0–100 scale, mean)	61.4 (16.0)	61.0 (12.0)
Pain episode duration (weeks, median)	52 (IQR: 17.5, 62.5)	52 (IQR: 16, 364)
Fear of movement (Fear Avoidance Beliefs Questionnaire: Physical Activity subscale) (0–24 scale, mean)	14.4 (6.4)	14.2 (6.6)
Activity limitation (Patient Specific Functional Scale) (0–100 scale, mean)	3.9 (1.7)	4.4 (2.4)
Activity limitation (Roland-Morris Disability Questionnaire) (0–100 scale, mean)	52.6 (19.9)	45.6 (26.4)
$AUD medication use per day at baseline	$1.03 ($0.91)	$0.96 ($0.75)
$AUD weekly income estimate based on gender and industry codes	$1467 ($265)	$1433 ($301)
Employment industry codes		
Professional, Scientific and Technical services	10 (26%)	5 (11%)
Health Care and Social Assistance	4 (11%)	10 (22%)
Agricultural, Forestry and Fishing	4 (11%)	5 (11%)
Administrative and Support services	4 (11%)	3 (7%)
Education and Training	2 (5%)	5 (11%)
Transport, Postal and Warehousing	2 (5%)	3 (7%)
Other categories	12 (32%)	14 (31%)

Data are mean (sd) or *n* (%) unless otherwise indicated

**Table 2:** Comparison of cost and clinical outcome measures between groups that were included in the economic evaluation

	Intervention	Control	Regression coeff (robust 95% CI)
$AUD lost productivity (total over 12 months)	$-6081 ($19, 627),	$-958 ($20,364)	$-5123 ($-10,174, $-72)
$AUD increase in medication use (total over 12 months)	$81 ($170)	$166 ($293)	$-85 ($-238, $68)
$AUD absolute imaging use	$52 ($191)	$66 ($169)	$-14 ($-125, $97)
$AUD absolute non-trial medical & therapies use	$137 ($232)	$170 ($285)	$-53 ($-105, $-0)
$AUD absolute trial medical & therapies use	$993 ($217)	$516 ($71)	$477 ($447, $508)
$AUD total cost (primary analysis)	$-4822 ($19,667)	$-40 ($20,278)	$-4781 ($-9748, $186)
$AUD total cost (sensitivity analysis)	$-4822 ($19,667)	$-557 ($20,277)	$-4265 ($-9221, $691)
Patient Global Impression of Change	8 Very much improved	2 Very much improved	
	15 Much improved	7 Much improved	
	10 Minimally improved	3 Minimally improved	
	5 No change	21 No change	
	0 Minimally worse	10 Minimally worse	
	0 Much worse	1 Much worse	
	0 Very much worse	1 Very much worse	
n (%) very or much improved on Patient Global Impression of Change (primary analysis)	23 (61%)	9 (20%)	0.41 (0.27, 0.54)
n (%) very or much or minimally improved on Patient Global Impression of Change (sensitivity analysis)	33 (87%)	12 (26%)	0.60 (0.46, 0.74)

Data are mean (sd)

**Fig. 2:**
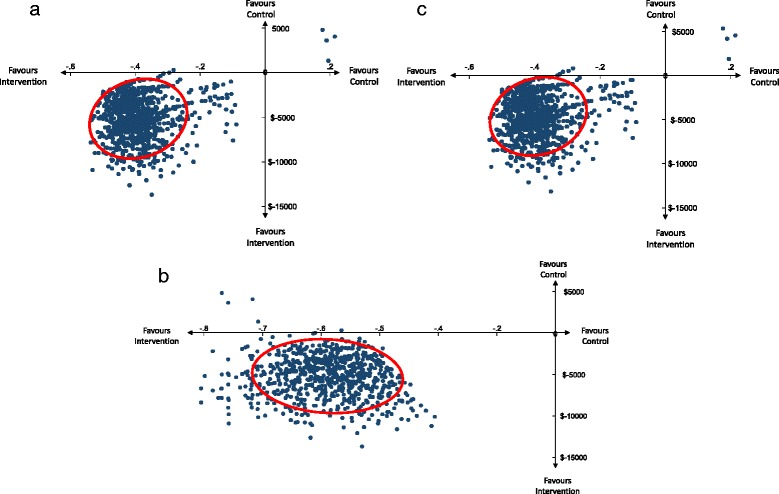
Cost-effectiveness plane with 95% confidence ellipse for incremental cost-effectiveness estimates for the primary analysis (**a**), first sensitivity analysis (**b**), and second sensitivity analysis (**c**)

The incremental cost-effectiveness of the intervention compared to the control condition for the primary analysis revealed that both total costs and clinical effects favoured the intervention group compared to the control. If there were 100 patients treated with the intervention, 41 more would have been ‘very much improved’ or ‘much improved’ than had those 100 patients been provided with the control condition. There would also have been a net saving of $478,100. Much of this saving however was driven by improved productivity costs. Thus it is not a direct saving to the health care system, rather, a gain for insurers, employers and society through participants being able to work more after receiving intervention with ViMove. The 95% confidence ellipse for the primary analysis revealed that the intervention dominated over the control condition (Fig. 2). Cost-effectiveness acceptability analysis was not pursued as the intervention dominated over the control condition with >99% certainty. Sensitivity analyses undertaken did not affect the conclusion of dominance of the intervention condition over the control condition, nor the degree of certainty of dominance of the intervention compared to the control condition being >99%.

## Discussion

This economic evaluation based on a pilot cluster randomised controlled trial indicates that use of a motion-sensor biofeedback system to augment guidelines-based care dominates (is both more effective and less costly) compared to guidelines-based care alone. It is important to be clear in this finding that the costs of providing the intervention was greater than providing the control in this study, but that the intervention group became more productive over the 12 month follow-up period and used fewer non-trial medical and therapy resources. This culminated in an overall saving to society, estimated to be $478,100 for every 100 patients treated to go with an additional 41 patients being classified as much or very much improved in relation to their low back pain. This finding of dominance was robust to the two key sensitivity analyses undertaken, where the costs of providing guidelines based care to the control group was eliminated, and where the threshold for classifying participants as being improved or not was changed.

The finding of dominance (both being less costly and more effective) with 95% confidence is particularly notable given the rarity of such a finding in this field. A review of economic evaluations for guideline endorsed interventions to reduce low back pain published in 2011 [8] included 26 studies, but identified only six studies where the intervention was thought to “dominate” over the control condition when considering cost-effectiveness [23–29]. In one, the follow-up period was only 3 months post-intervention and did not include costs of medical services outside the trial protocol [23]. Another did not formally undertake an incremental cost effectiveness analysis [24, 25]. Another did not examine the 95% confidence interval ellipse surrounding the incremental cost-effectiveness estimate. This was important for this study given the highly skewed cost data they reported [26]. Of the studies that examined the 95% confidence ellipse that surrounded the incremental cost effectiveness estimate, none demonstrated dominance with 95% confidence [27–29].

Some caution should be employed when using these findings to guide clinical decision making and policy formation. Data used to build this economic evaluation were derived from a single, pilot (though of reasonable size) randomized trial. It is the nature of “concurrent economic evaluations” that any design-related issues or concerns of bias or imprecision in effect size estimates relating to the original randomized trial equally apply to the economic evaluation. In the same way that authors of the original trial recommended that a fully powered trial was warranted to be clear on the effectiveness of this intervention [10], further research is also required to confirm the results of this economic evaluation. This trial has demonstrated that the motion-sensor biofeedback approach appears to be a viable intervention from both a clinical and economic perspective, and that future research using this approach should be a priority.

Productivity costs were a key driver of the outcome of this economic evaluation. This was not surprising given that productivity costs are the key driver of burden of disease estimates in this condition [3]. Our approach to calculating productivity was relatively indirect in that we estimated income based on occupation and industry rather than directly capturing income from the participant. We also estimated presenteeism by using a linear extrapolation of the participants reported ability to perform their occupation. Using indirect methods to calculate productivity in economic evaluations conducted alongside clinical trials is common [30] and is often necessary given the threat to broader trial viability that more intensive data collection methods for these outcomes may create. These concerns may have introduced random error into our productivity estimates, but as these categorisations were undertaken by an investigator blinded to group allocation status (KB) we do not believe this would have created a systematic bias between groups that would have had a substantial effect on our results or conclusions. We also ignored the potential for productivity losses in one individual to negatively affect co-workers’ productivity in case of team-dependent production. The productive output of a full team can be jeopardized by one member’s illness [30]. This is more likely to have affected the group with more productivity losses (the control group in our case), which would make our cost effectiveness estimate more conservative.

Further research in this field is clearly indicated. First, to conduct a larger randomized trial and economic evaluation. Second, to understand potential mechanisms of action between the motion-sensor biofeedback intervention and improved productivity. Such research should consider whether it is purely changes in pain and function that drive improved occupational performance, or whether there are additional psychological benefits from wearing the device. The function of the motion-sensor biofeedback system to be able to alert the wearer when they remain in a particular position for too long or if they move in a way that may compromise their musculoskeletal health may reassure them and make them feel as if they can perform their work with less risk of injury. This may enhance their willingness and motivation to participate in their occupation. Understanding potential mechanisms of action may be useful for refining treatment protocols that optimise the value of this approach.

## Conclusion

Low back pain continues to have a major impact on occupational productivity around the world, despite years of research to address this problem. The motion-sensor biofeedback treatment approach investigated in this research appears to be both more clinically effective and economically efficient than guidelines based care alone. This approach appears to be a viable means to manage low back pain that may enhance productivity. Further research in this area should be a priority as these data have been drawn from a pilot randomised trial.

## Abbreviations

CI: Confidence interval
sd: Standard deviation
